# Health Promotion Practice Among Older Persons: A Nordic Multi-Professional Focus Group Study Exploring what It Is and How It Could be Achieved

**DOI:** 10.1177/07334648221082021

**Published:** 2022-03-27

**Authors:** Emilia W.E. Viklund, Johanna Nordmyr, Greta Häggblom-Kronlöf, Anna K. Forsman

**Affiliations:** 1 Faculty of Education and Welfare Studies, Health Sciences, 421531 Åbo Akademi University, Vaasa, Finland; 2 Institute of Neuroscience and Physiology, Section for Health and Rehabilitation, The Sahlgrenska Academy, 195564 University of Gothenburg, Gothenburg, Sweden; 3 Centre for Ageing and Health–Agecap, 195564 University of Gothenburg, Gothenburg, Sweden

**Keywords:** health services, occupational groups, healthy ageing, ageing, focus groups, Finland, Sweden

## Abstract

The growing ageing population in the Nordic region calls for increased focus on health promotion work. To enhance multi-professional understanding and further develop strategies for promoting healthy ageing, it is vital to consider the perspectives of those working with health promotion. The aim of this study was to explore a wide spectrum of practitioners’ experiences of community-level health promotion targeting older adults in Finland and Sweden. Nine focus group interviews (34 informants) were conducted in 2019–2020. “Seeing the person” emerged as the ideal for health promotion targeting older adults, but this ideal was not always realized in current practice. Barriers related to organizational structures and the practitioner role were identified. However, work methods connected to user involvement and technology-based tools were considered key facilitators, enabling tailored health promotion initiatives.

## Introduction

The growing and increasingly heterogeneous ageing population is a megatrend of our times (Sitra, 2020) and recent forecasts presume that over 8% of the Nordic population will be 80 years or older in 2040 (Nordic Welfare Centre, 2020). The growing ageing population is often considered to be a negative social phenomenon, described in terms of expected social and healthcare sector burden and related public expenditure (Arai, et al., 2012). However, longer lives and a larger share of older persons within populations can also provide opportunities for both individuals and society (WHO, 2017). In order for later life to constitute a period marked by opportunities to lead life as desired, health and well-being are seen as key resources (WHO, 2017), and therefore, the promotion of health and well-being among older adults should be considered important areas of action in the forthcoming years.

Health promotion is defined as “the process of enabling people to increase control over, and to improve, their health” (WHO, 1998, s.1) and covers diverse methods and approaches at local, national, and international levels and is fundamental in the clinical work of various professions and voluntary groups alike (WHO, 1984). Recently, international health promotion strategies targeting older adults have centered around the concept of healthy ageing—the creation of environments and opportunities that enable persons to lead later life as they desire (WHO, 2020). In order to promote healthy ageing for all, strategic work on increasing person-centered approaches in primary health services, responsive to the needs of the older adults, has been warranted along with multi-sectoral collaboration and meaningful engagement of older persons (WHO, 2020).

Utilizing semi-structured interviews, Bryant et al. (2001) explored older persons’ perceptions of what constitutes healthy ageing, which was described as going and doing something meaningful. Similar results were found in another study (Nordmyr et al., 2020) focusing on the oldest-olds’ perceptions of mental well-being, which was conceptualized in a four-dimensional model consisting of activities; capability; orientation; and connectedness. From the practitioners’ perspective, health promotion targeting older persons in residential care should entail promoting self-determination, autonomy, and social integration (Marent, et al., 2018) while improving quality of life is the number one goal when working with older persons with multiple chronical conditions (Ploeg, et al., 2019). Additionally, nurses working in various healthcare settings (Kemppainen et al., 2012) have emphasized the need for taking the individual’s specific needs into account as well as suggesting empowerment to be one of the most important principles of health promotion.

However, the current literature base covering practitioners’ experiences of health promotion practice reveals a gap between the practitioners understanding of health promotion in theory and its practical implementation, where, for example, the organizational structures and culture impacts practice (Marent, et al., 2018). Nurses highlighted lack of resources and time as factors hindering them from incorporating health promotion activities within their clinical work (Kemppainen et al., 2012). In line with these findings, practitioners working within home care services perceived health promotion as difficult to integrate into their working routines as they experienced that other tasks were prioritized, such as solving acute problems and emergencies (Karlsson, et al., 2020).

Taken together, experiencing health and well-being are important resources at every stage of life and increased attention needs to be paid towards promoting health and well-being among older adults. In order to develop strategies for promoting healthy ageing further, it is vital to consider the perspectives of those working with health promotion. While the studies highlighted above focused on specific professional groups (nurses) or contexts (residential age care or home care), it is motivated to conduct similar enquiries focusing on a multi-professional perspective given that inter-sectoral and multi-professional collaboration constitutes key principles in health promotion (Corbin, 2017).

The study at hand aimed to gather multi-professional knowledge and experiences regarding community-level health promotion targeting older persons in the Nordic context. The study explored a wide range of practitioners’ perspectives on what health promotion targeting older persons should entail and related barriers and facilitators for such health promotion practice.

## Methods

### Data collection

The framework for reporting qualitative studies (COREQ) by Tong et al. (2007) was followed during the drafting of the paper and a completed checklist is included as supplementary material in order to ensure trustworthiness.

Focus group interviews are considered a suitable data collection method for gaining an understanding of a phenomenon from the perspective of the participants themselves (Hennink & Leavy, 2014). The synergy and interaction that the group discussions foster uncovers a range of experiences and perspectives, which can enrichen the data (Carey & Asbury, 2016) compared to conducting individual interviews. Focus group interviews have been conducted with a wide range of different population groups and in various settings (Carey & Asbury, 2016).

The data consist of nine focus group interviews conducted in Ostrobothnia (Finland) and in Västra Götaland (Sweden) during October 2019 to January 2020. The focus group interviews lasted from 1 hour up to 2 hours, dependent on the group size. An interview guide (Supplementary material), consisting of four themes, was used to facilitate the discussions between the informants. The authors, who also conducted the interviews, jointly developed the interview guide and the questions were chosen in order to capture the practitioners’ perspectives and experiences of health promotion targeting older adults. All authors had substantial experience in focus group methods and all of the interviews were conducted by at least one of the authors. The lead author participated in seven out of nine of the focus groups in order to ensure consistency and the other authors participated as co-moderators.

### Study Setting and Informants

Health and social services aiming to promote health and well-being among older adults are provided by a wide spectrum of practitioners, as well as volunteers, working within different types of organizations (public sector and third sector, that is, non-governmental and non-profit-organizations and associations) and representing different professions and educational levels.

When recruiting informants to a focus group interview, heterogeneity and homogeneity are key (Kreuger & Casey, 2009). Focus groups are not primarily aiming for consensus-building regarding a topic—breadth and richness of experiences and perspectives are usually strived for with the method (Carey & Asbury, 2016). Therefore, the design may include a purposeful selection of informants representing a range of experiences related to the study topic. In total, 34 practitioners participated in the focus group interviews (see Table 1).

**Table 1.** Information about the focus groups and the participating informants.

Descriptive information	N (%)
Focus groups	9
Informants (participants per group)	34 (mean 3.78, range 2–6)
Gender	
Women	28 (82.4)
Men	6 (17.7)
Study region	
Österbotten (Finland)	17 (50)
Västra Götaland (Sweden)	17 (50)
Sector	
Public	24 (70.6)
Third	10 (29.4)
Work descriptions	
Coordinator for activities and services for older persons	8 (23.5)
Physiotherapist	5 (14.7)
Counsellor/advisor	5 (14.7)
Home care worker	3 (8.8)
Occupational therapist	2 (5.9)
Nurse	2 (5. 9)
Physician	1 (2.9)
Social worker	1 (2.9)
Deacon	1 (2.9)
Head of home care unit	1 (2.9)
Head of third sector association	1 (2.9)
Head of the department for senior services	1 (2.9)
Superintendent responsible for clients’ living facilities and practical arrangements surrounding social activities	1 (2.9)
Physiotherapist assistant	1 (2.9)

Information about the study was distributed through email or phone via the head of organizations that provided services and activities aiming to promote health and well-being among older persons in the study regions. Eligibility criteria for study participation included recognizing oneself as working with community-level health promotion work and with community-dwelling older adults, as well as work experience of at least 1 year. In order to obtain diverse experiences from the group discussions, the authors purposefully included practitioners from different municipalities, different kinds of organizations, and with different professions and work descriptions. The group composition was partly based on the informants’ place of residence and partly with a multi-professional approach in mind, striving for having practitioners with different kinds of work roles in each focus group. However, the goal of all of the groups being multi-professional did not fully succeed in case of two groups because of last minute dropouts due to illness.

### Ethical Considerations

The ethical principles of the Helsinki Declaration (World Medical Association, 2013) were carefully followed throughout the study and drafting of the article. Written informed consent was obtained from the participants, who were informed about their right to withdraw from the study at any time, and that their information would be kept strictly confidential. The study did, however, not require formal ethical assessment according to the national boards of research integrity and ethics, as the study informants participated in the study in a professional capacity and the study did not pose a threat to their health, physical integrity, or safety.

### Analysis

**Figure 1.**
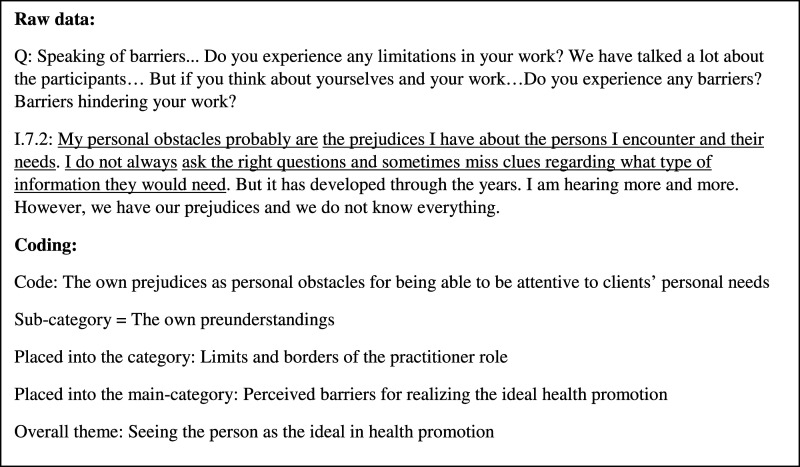
Illustration of the coding process.

The interviews were recorded and transcribed manually verbatim by the authors, resulting in 151 pages of text. Inductive qualitative content analysis, described and exemplified by Kyngäs (2020), was used as a guide for the exploratory analysis of the transcribed data. The first step of the data analysis was to listen to the recorded interviews and read the transcript several times. Thereafter, codes were created by writing down sentences describing the content of the transcripts, hence reducing the data. The codes were in the next step of the analysis compared to each other and based on similarities and differences grouped together, forming sub-categories. The sub-categories were subsequently reorganized and formed into groups based on reflecting similar content, creating the categories—the perceived barriers and facilitators. Furthermore, “seeing the person” was recognized as something that recurred throughout the transcripts and was seen as an overreaching theme (see Figure 1 for an illustration of the coding process). The analytical steps were redone several times until the authors agreed upon the structure. The sub-categories, categories, main categories, and the theme that the data analysis generated are displayed in Table 2.

**Table 2.** Theme, main categories, categories, and sub-categories.

Overall theme: Seeing the person as the ideal in health promotion		
Main categories (two)	Categories (four)	Sub-categories (eight)
Perceived barriers for realizing the ideal health promotion	Limits and borders of the workplace	Strict organizational structure and guidelines Inflexible working routines
	Limits and borders of the practitioner role	The own preunderstandings The ethical competence
Perceived facilitators for realizing the ideal health promotion	Means for crossing the borders of the workplace	Assistance by digital technology Accessing a wide range of activities through online platforms
	Means for crossing the borders of the practitioner role	Involving older persons in health promotion initiatives The future of user-driven health promotion initiatives

### Findings

#### Seeing the Person as The Ideal in Health Promotion

The importance of seeing the older adults as unique persons was identified as the overall theme, pervading all the focus group discussions. The older adults were discussed in terms of being diverse persons with individual needs and preferences, which should be kept in mind when it comes to promoting health and well-being:

We cannot talk about older adults as a homogenous group, it would be as wrong as it is to talk about women as a homogenous group. There is a wide spectrum of older persons, there are for example both persons who are in good health and fully capable of taking care of themselves, as well as persons who are receiving palliative care in nursing homes. [I.9.6]

Thus, the subjective perspective of the older person was perceived as central when it comes to health promotion practice. Ideally, the person’s own needs and preferences should be the starting point in all work:

As practitioners, we know what health is and we have our perspectives regarding what promotes the older persons’ health. But the thing is, the older persons do not necessarily share the practitioners’ view of health. Therefore, the older person’s own perspective of health should be the starting point. [I.4.1]

**Figure 2.**
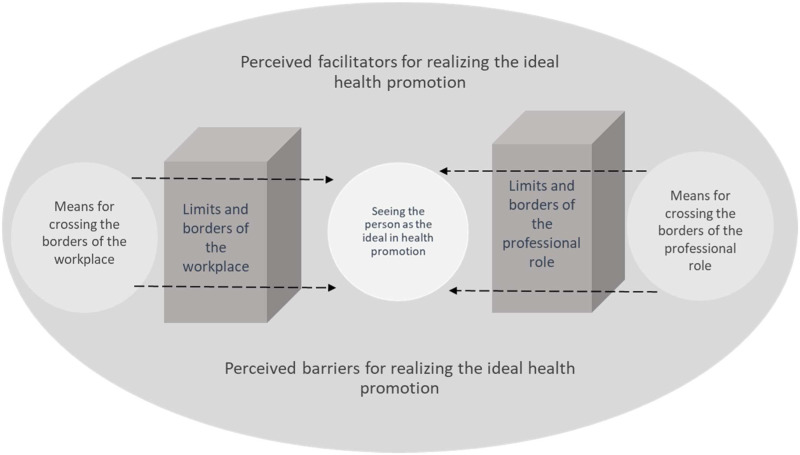
Illustration of the perceived barriers and facilitators for ideal community-level health promotion practice among older persons.

Despite the high agreement between the informants regarding seeing the person as the overall ideal in health promotion practice, the reality did not always allow the informants to work according to these ideals. The main categories were identified as the perceived barriers and the perceived facilitators for realizing the ideal health promotion practice. Figure 2 is an illustration of the study findings.

### Perceived Barriers for Realizing the Ideal Health Promotion Practice

The informants perceived barriers in everyday practice, factors that currently hindered them from being able to work in a way that was attentive to the needs and preferences of the older persons. Two types of barriers were identified, connected to the limits and borders of the workplace, and of the practitioner role.

### Limits and Borders of the Workplace

The limits and borders of the workplace encompassed strict organizational structures and guidelines and inflexible working routines.

The opportunity to be flexible and spontaneously respond to fluctuating preferences among the clients was identified as a key factor for succeeding with tailoring health promotion initiatives to meet the older persons’ needs. However, the organizations’ existing routines and guidelines did not correspond with this key factor—they were perceived as often being inflexible and insensitive to unique needs and preferences. Consequently, the informants sometimes decided to go against the guidelines of the organization to support the client in a way that is best suited to the situation.

It (the system/organization) is just so terribly rigid and difficult to follow. From my point of view, it can be very difficult to reach the person if I am not allowed to work around it. [...]. I.6.1: So please do not report from which municipality this is. *laughter*. [...]. [I.6.4]

The narratives contained descriptions of a perceived dissonance between the universal, standard, guidelines and working routines developed by the organization and the needs of the older persons. Informant I.6.1 described recent attempts within the organization to digitize services and activities as situations where gaps between the new working routines and the preferences of the older persons became apparent:

I feel that we get immediate feedback from our guests [the older persons] on what works and what doesn’t. One example is the recent digitalization of everything in our organization. We want to be a part of the digital world and have taken IT-classes focused on older persons and so on, but there is still a lot of older persons who do not use the internet. More than those in charge seem to think. “Really, is that true?” Yes. There are still many who do not use the internet. Just such things, you know.[I.6.1]

Furthermore, having adequate time resources was perceived as a condition for being able to see the person, which was not being fulfilled within the frames of the current working routines. One example of a routine experienced as resource-intensive, taking focus and time from the encounters with the older persons was the increased use of digital tools. A mutual worry regarding how the person-to-person interaction might be replaced by technology in the future was identified in the data analysis. For instance, informant I.1.3 already felt that the digital tools have changed the notion of her everyday work—far from the desired and expected practice:

One is increasingly becoming more of a coordinator for others when the computer-based work is taking more and more time. It means that you have less time to meet with the persons yourself… I do not like this development, because you expect to be present to help and support persons, not working with computer programs. [I.1.3]

### Limits and Borders of the Practitioner Role

The limits and borders of the practitioner role were connected to the informants’ preunderstandings and ethical competence.

The importance of practitioners being aware of the fact that the clients’ outlook on health is not necessarily equal to the practitioners’ perspectives on what contributes to healthy ageing was highlighted frequently. The practitioner perspective was perceived to sometimes restrain the informants’ opportunities to be attentive to the clients’ needs and preferences. Education and personal and professional experiences formed a lens through which the practitioners saw the older persons’ situation and this lens could filter out important indicators of the clients’ perspective:

My personal obstacles probably are the prejudices I have about the persons I encounter and their needs. I do not always ask the right questions and sometimes miss clues regarding what type of information they would need. But it has developed through the years. I am hearing more and more. However, we have our prejudices and we do not know everything. [I.7.2]

Supporting the older persons’ viewpoint and preferences regarding health and well-being was a self-evident part of the ideal health promotion, but was perceived as complicated to realize in practice. Ethical dilemmas connected to ignoring the practitioner view of health in favor of listening to the persons’ own preferences were identified. The informants’ ethical competence was especially questioned in situations where the practitioners had reason to suspect that the clients were not able to make informed decisions regarding their own health due to mental or cognitive problems. The informants further discussed the personal challenges of accepting the older persons’ preferences, even if being in opposition to the practitioners’ perspective:

A person with a dementia diagnosis wanted to walk home from our daytime activities instead of receiving the usual home transportation. We discussed the practitioners’ worries regarding the situation and finally agreed that the person will be able to walk home, but that the personnel will call him in the afternoon. After a few weeks, the person came to me and said that he felt that the situation was difficult, because he felt that he had to hurry back home to answer the phone call. […]. We need to remove our own worries and feelings from the decisions we make. [I.6.4]

### Perceived Facilitators for Realizing the Ideal Health Promotion Practice

The everyday practice of the practitioners was not only marked by obstacles—the informants also shared views on how the older persons’ personal needs and preferences could become more prominent in current and future health promotion practice. Two kinds of facilitators were generated from the data—means for crossing the borders of the workplace and means for crossing the borders of the practitioner role.

### Means for Crossing the Borders of the Workplace

The means for crossing the borders of the workplace were assistance by digital technology and accessing a wide range of activities through online platforms.

The development associated with the digitalization of the social and health care sector was perceived as a blessing in disguise. The narratives contained ideas about how technology is (and could be even more in the future) contributing to the realization of ideal practice. Technology could potentially assist the practitioners with some of the technical or routine work tasks to larger extents than today. In this manner, technology might release more time to concentrate on the older persons and their needs:

Of course, there are also benefits. As she mentioned, being able to bring with you the medicines, already dispensed for two weeks ahead, means that you can spend your time speaking with the client instead of focusing on the medications. Other things might be brought up if you are not dispensing the medication in front of the client. [I.2.1]

Digital technology was also identified from the narratives as a means for offering various services and activities for diverse interests and needs. Online platforms could provide access to a more versatile range of activities, which do not necessarily have to be delivered by the local municipality. By accessing online services and activities nationally but also internationally, there is no need to fit all the older persons in the municipality to one or a few local health promotion initiatives:

1.4.3: I think that if you do not like being around other people.. I.4.2: Or if you do not like being alone but neither in a group.. I.4.3: Then I think technology is a good alternative. With [online] exercise routines for example, you can experience that you are doing something good for yourself but do not have to participate in group-based activities. [I.4.3, I.4.2].

### Means for Crossing the Borders of the Practitioner Role

The means for crossing the borders of the practitioner role included involving older persons in health promotion initiatives and the future of user-driven health promotion initiatives.

The importance of involving older persons in the initiatives was frequently mentioned in relation to successful health promotion. By increasing the role of the older persons and encouraging them to take an active part by giving them the opportunity to influence the content of the activities and services, the informants perceived that the barriers connected to preunderstandings could be bridged. Various methods, for example, evaluation questionnaires, reference groups and advisory boards were suggested to be tangible tools for ensuring needs-based activities and services:

I experience that our services and activities have become more person-centered since we started with user involvement. We organize boards for our guests [older persons] and I have witnessed that our guests have taken a larger role in the activities ever since. I think it is rewarding. It is health promotion. We also have activities where the guests are being served coffee and it is cozy too, but for me user involvement is a big thing. Feeling that this is my activity. [I.6.1].

The informants further expected that the older persons will play a large role in producing and delivering health promotion services and activities in the future. The future vision entailed activities and services becoming more peer-to-peer based, a development which would diminish practitioners’ impact on the content of the activities and services. Digital technologies, such as social media, were also recognized as potentially becoming even more helpful tools for empowering older persons in creating their own health promotion initiatives, in addition to, for example, new housing options, equipped with spaces for social gatherings. The envisioned future of the older persons as the main driving force behind the health promotion activities and services was noted by the informants:

I.1.2:I expect that the future will bring more [older] volunteer workers and that they will be leading a larger part of the group-based activities. I.1.4: We [the practitioners] will become coordinators and the volunteers or peers will be taking care of the activities. Because there is a lot of people feeling lonely. I.1.3: There would be someone [practitioner] who initiate the activities first and then the volunteers could take over and continue the activities. [I.1.2, I.1.4, I.1.3]

## Discussion

“Seeing the persons,” with unique needs and preferences, was highlighted by the study informants as the ideal in health promotion practice among older adults. Similar ideas connected to acknowledging the uniqueness of persons are found within person-centered care, an approach that advocates for seeing the patients or clients as unique persons and active partners (e.g., Edvardsson, 2015). To consider the diversity of the older adults as well as the importance of tailoring initiatives to the individual persons’ own desires can also be grasped from international policies connected to ageing and health, such as the decade of healthy ageing (WHO, 2020). However, despite the broad recognition of the vital role of being attentive to older persons’ needs and preferences, the findings suggest that there is a discrepancy between what is being perceived as ideal and the actual, realized, practice.

The practitioners experienced that existing organizational structures, guidelines and working routines, limited their opportunities to “see the person,” which is consistent with the experiences of both practitioners (Olsen, et al.. 2019; Moore, et al., 2017) and older home care clients’ (Dunér, et al., 2019)-older persons’ preferences are not being considered to the extent necessary for ideal practice. Additionally**,** Janssen et al.. (2021) found that occupational therapists working with community-based health promotion experienced a challenge with taking older persons’ perceptions of health and related preferences into account because of health promotion initiatives typically being developed to meet the need of groups of people, not individuals. Moreover, the strict guidelines might be designed to prescribe standardized services to groups of older persons simultaneously as they ensure that the practitioners are keeping up with tight working schedules in order to meet the organizational demands for efficiency and productivity (Olsen, et al.., 2019) but might not adhere to the client’s personal needs and preferences.

However, the practitioners in this study suggested that digital technologies could become tangible assistive tools to create more flexible working schedules. For instance, by technology overtaking some of the more routine-based work tasks (such as medication dispensing or administration), the practitioners could concentrate on “seeing the person.” Similar assistive potentials with digital technologies, such as robots, within health-related services, and heavy care activities have also been recognized in previous studies among healthcare personnel (e.g., Turja, et al., 2018). On the other hand, worries that the future working routines of the practitioners will become more technology-centered instead of person-centered, were also generated from the data analysis, in line with the findings of previous studies (Pekkarinen, et al., 2020; Segercrantz & Forss, 2019; Frennert & Baudin, 2019). The worries connected to increasingly digitized health services and activities are reasonable with regards to both the digital divide that still exist among some groups of older adults (Czaja, 2021) and the practitioners own working environment. Persons representing older age cohorts and with more health conditions are less likely to use smartphones even though they could benefit the most from using this kind of technology (Mohlman & Basch, 2021). Additionally, the entrance of various digital tools and services has not only facilitated practice as intended and the discrepancy between the visions of digital technologies as facilitators and the constrained reality has been connected to deficient implementation processes (Frennert, 2020). Practitioners are seldom actively involved in the implementation nor the development process of digital services (Frennert, 2020), meaning that their expertise and contextual knowledge are not being captured in terms of how digital technology best could fit into everyday practice. Increased involvement of practitioners in the development and implementation of both online and offline health promotion initiatives could perhaps untangle some of the barriers for ideal health promotion practice.

Additionally, actively involving older persons in both the planning of and the realization of activities was suggested as a means for reducing the impact of practitioners’ own preunderstandings on the practice in this study. Jönsson (2013) describes how older persons often are portrayed within care settings as having more basic and modest needs and preferences than younger generations. In a recent review of municipal guidelines for social and health care services targeting older adults, Möllergren (2021) found traces of this ageing stereotype. Furthermore, the suggestion of increased user involvement as a facilitator for ideal health promotion practice is supported by scholars within the field of technology development, as actively involving the end-users throughout the design process is recognized as an effective means for creating technological products in line with the target group’s needs (Fischer et al., 2020) as well as for combating ageing stereotypes (Frennert & Östlund, 2014). However, there is a lack of research on how to work with user involvement within ageing and health and especially when it comes to involving more vulnerable older persons (Berge, et al., 2020). This type of information would certainly be essential in order to co-create future inclusive and diverse health promotion initiatives—attentive to the needs and preferences of heterogeneous older populations.

Taken together, in order to meet the demands of future ageing societies, attention needs to be paid towards the growing diversity in the ageing population. Finding the balance between standardized working routines, ensuring the same high-quality and equal health-related services and offering tailored initiatives supporting unique needs—focusing on equity, is essential when it comes to forming the future health promotion for older persons. This includes creating more flexible working routines and organizational structures where the practitioners are allowed to be attentive to and respond to the unique and fluctuating needs of older persons, for example, by increasing the use of digital technology within the daily working routines and added online alternatives for older persons. Additionally, older persons as well as practitioners should increasingly be involved in the creation and implementation of new working routines and health promotion activities and services.

### Strengths and Limitations

To establish trustworthiness in terms of the credibility, dependability, confirmability, authenticity and transferability (Kyngäs, et al., 2020) the authors have made an effort to carefully describe the sample as well as the study procedure without jeopardizing the informants’ anonymity. Furthermore, the results are illustrated with tables, figures and quotations from the interviews to allow for data coding transparency.

The group dynamic and the possibilities that focus group interviews provide for the informants to influence each other’s thoughts and ideas can be viewed as both a benefit and a challenge (Leung & Savithiri, 2009). Finding a balance between sample homogeneity and heterogeneity is a key for fostering interactive focus group discussions and for gathering a broad and rich data material (Kreuger & Casey, 2009). Thus, heterogeneity in terms of the informants’ work descriptions, educational levels, and professional backgrounds as well as the fact that the study was conducted in two fairly different regions in two countries can be seen as both a limitation and a strength. A high degree of heterogeneity can complicate the discussions, and therefore, some researchers advocate for keeping separate groups for gender, age groups, professions, and educational levels (Carey & Asbury, 2016). The diversity of the sample may have contributed to some lack of depth and that informants might have been hesitant to share their actual experiences if it departed from the experiences of the rest of the group, especially if other group members had higher educational level and a more authoritarian role. On the other hand, the interaction and collaboration between different professions and sectors are seen as essential elements in health promotion (Leppo, et al., 2013). Additionally, keeping the groups homogenous with regards to professions can be seen as narrow in terms of capturing varying perspectives and experiences (Carey & Asbury, 2016). Moreover, the study holds no ambition to compare health promotion targeting older adults between the countries or regions or analyze the perceptions according to different work descriptions or educational level. The multi-professional approach contributed to a broad and rich data material on the practitioners’ perceptions regarding what health promotion targeting older persons should entail and barriers and facilitators for ideal practice alike.

Finally, the findings are a result of interpretations made by the researchers, and the fact that they are experienced health promotion scientists- bringing their preunderstandings to the study- constitutes potential biases for the study at hand that cannot be overlooked.

## Supplemental Material

sj-pdf-1-jag-10.1177_07334648221082021 – Supplemental Material for Health Promotion Practice Among Older Persons: A Nordic Multi-Professional Focus Group Study Exploring what It Is and How It Could be Achieved Click here for additional data file. Supplemental Material, sj-pdf-1-jag-10.1177_07334648221082021 for Health Promotion Practice Among Older Persons: A Nordic Multi-Professional Focus Group Study Exploring what It Is and How It Could be Achieved by Emilia Viklund, Johanna Nordmyr, Greta Häggblom-Kronlöf and Anna Forsman in Journal of Applied Gerontology
