# Supplementary material for: Health Promotion Practice Among Older Persons: A Nordic Multi-Professional Focus Group Study Exploring what It Is and How It Could be Achieved
Source: J Appl Gerontol. 2022 Mar 27;41(7):1665–74. doi: 10.1177/07334648221082021 (PMC9210104; doi:10.1177/07334648221082021)
Supplement: sj-pdf-1-jag-10.1177_07334648221082021 – Supplemental Material for Health Promotion Practice Among Older Persons: A Nordic Multi-Professional Focus Group Study Exploring what It Is and How It Could be Achieved [file sj-pdf-1-jag-10.1177_07334648221082021.pdf]

Supplementary material

Supplementary file 1

COREQ 32-item checklist for interviews and focus groups (Tong, Sainsbury & Craig, 2007)

**Research team and reflexivity**		
1.	Interviewer/facilitator	The authors 1, 3 and 4 conducted the interviews.
2.	Credentials	Author 1 = MSocSc<br>Author 2 = PhD<br>Author 3 = PhD<br>Author 4 =DrPH
3.	Occupation	Author 1 = Doctoral student<br>Author 2 = Project researcher<br>Author 3= Associate Professor<br>Author 4= Associate Professor
4.	Gender	All of the researchers are female.
5.	Experience and training	Author 1 = In doctoral training<br>Author 2 = Has participated in planning and conducting multiple focus group studies.<br>Author 3= Had led national research projects focusing on older persons and health in everyday life and health promotion interventions and RCT studies.<br>Author 4 = Has led several national and international research projects focusing on health promotion contexts and approaches.
**Relationship with participants**		
6.	Relationship established	The researchers were not employed by any of the organizations that the practitioners represented. The recruitment of informants report no bias grounded on dependency issues.
7.	Participant knowledge of the interviewer	All of the informants knew which organizations the researchers represented and their research interests in older adults' health and wellbeing.
8.	Interviewer characteristics	In the strengths and limitations section, the researchers' preunderstandings as health promotion scientists are highlighted as potential bias.
**Theoretical framework**		
9.	Methodological orientation and theory	Exploratory, inductive, qualitative content analysis.

**Participant selection**		
10.	Sampling	Purposeful sampling
11.	Method of approach	Email and Phone
12.	Sample size	34
13.	Non-participation	Ten persons declined or did not respond to the invitation. One person dropped out from the study due to being ill when the focus group were held.
14.	Setting of data collection	The focus groups were conducted in various places and settings, such as the universities, the informants' workplaces and city halls. The focus group location was chosen in order to be convenient and easily accessed by the informants. One informant also participated in the group discussions via Skype, due to sudden logistical challenges.
15.	Presence of non-participants	-
16.	Description of sample	Practitioners, representing different organizations, educational levels and professions, working with health promotion targeting community-dwelling older adults, with at least one year of work experience. Description of the focus groups and the informants are displayed in Table 1.
**Data collection**		
17.	Interview guide	An interview guide encompassing broad themes and related questions was used (see supplementary material). The interview guide was not pilot tested, but was continuously discussed within the research group.
18.	Repeat interviews	-
19.	Audio/visual recording	Audio recording was used to collect data.
20.	Field notes	The interviewers made notes during the focus groups.
21.	Duration	One and up to two hours, dependent on the group size.
22.	Data saturation	Data saturation was discussed among the researchers after the 8th interview and the researchers decided to not recruit more focus groups than the nine groups that were already appointed.
23.	Transcripts returned	-
**Data analysis**		
24.	Number of data coders	One researcher conducted the initial coding of the data and all

		four worked with generating the categories and theme.
25.	Description of the coding tree	Table 2 displays the codes, sub-categories, main-categories and the theme and Figure 1 is an illustration of the coding process.
26.	Derivation of themes	The theme, main categories and sub-categories were derived from the data.
27.	Software	Microsoft Word
28.	Participant checking	-
**Reporting**		
29.	Quotations presented	The questions are presented in supplementary files. Each quotation is identified by reporting the informants' codes.
30.	Data and findings consistent	The authors have made an effort to keep the findings close to the original data and the authors who conducted the interviews did recognize the discussions in the final findings.
31.	Clarity of major themes	The major themes are described in text as well as in Table 2 and Figure 1.
32.	Clarity of minor themes	The minor themes are described in the text as well as in the Table 2 and Figure 1.

Supplementary file 2

Interview guide

Original language of the interview guide was Swedish. The guide was translated into English by the authors.

1. Theme: Everyday work

Tell us about your work. What are your main areas of responsibility and main tasks?

Could you tell us about your work right now, focusing on the positive experiences?

Do you experience any challenges? What could be improved?

2. Theme: Health promotion

In your view, what is health promotion? And health promotion work? Can you give any examples from your own work?

What do you think about user involvement, i.e. involving the target group, in this case the older adults, in the activities. Can you give any examples from your own work?

3. Theme: The future

With the discussions of today in mind, if you look 20 years ahead in time, what do you think the situation will look like then? In relation to your work and your work tasks.

If themes related to technology and innovation have not been addressed in the discussions: What is your view on technology in relation to health promotion in later life? In relation to your work tasks? Now and in the future.
